# Human milk oligosaccharides modify the strength of priority effects in the *Bifidobacterium* community assembly during infancy

**DOI:** 10.1038/s41396-023-01525-7

**Published:** 2023-10-10

**Authors:** Martin F. Laursen, Henrik M. Roager

**Affiliations:** 1 National Food Institute, Technical University of Denmark, Kgs. Lyngby, Denmark; 2 Department of Nutrition, Exercise and Sports, University of Copenhagen, Frederiksberg C, Denmark

**Keywords:** Ecosystem ecology, Microbial communities, Next-generation sequencing, Bacteriology

## Abstract

Despite the significant role of the gut microbiota in infant health and development, little is known about the ecological processes determining gut microbial community assembly. According to ecology theory, the timing and order of arrival of microbial species into an ecosystem affect microbial community assembly, a phenomenon termed priority effects. *Bifidobacterium* species are recognized as highly abundant early colonizers of the infant’s gut, partly due to their ability to selectively utilize human milk oligosaccharides (HMOs) from breast milk. However, the role of priority effects in *Bifidobacterium* community assembly remains unclear. Here, we investigated the *Bifidobacterium* community assembly in the gut of 25 breastfed Danish infants longitudinally sampled throughout the first 6 months of life. Our results showed that the breastfed infants were often initially, but temporarily, dominated by suboptimal HMO-utilizing *Bifidobacterium* taxa, such as *B. longum* subsp. *longum*, before more efficient HMO-utilizers such as *B. longum* subsp. *infantis*, replaced the first colonizer as the dominant *Bifidobacterium* taxon. Subsequently, we validated this observation using gnotobiotic mice sequentially colonized with *B. longum* subsp*. longum* and *B. longum* subsp. *infantis* or vice versa, with or without supplementation of HMOs in the drinking water. The results showed that in the absence of HMOs, order of arrival determined dominance. Yet, when mice were supplemented with HMOs the strength of priority effects diminished, and *B. longum* subsp. *infantis* dominated regardless of colonization order. Our data demonstrate that the arrival order of *Bifidobacterium* taxa and the deterministic force of breast milk-derived HMOs, dictate *Bifidobacterium* community assembly in the infant’s gut.

Inadequate development of the infant gut microbiota has been linked with multiple adverse conditions such as asthma and allergies [1, 2], autoimmune diseases [3, 4], inflammatory bowel diseases [5], and poor neurological development and growth [6]. However, our understanding of the processes that govern microbial community assembly in the gut during infancy remains incomplete [7]. According to ecological theory, timing and order of arrival of species into a specific ecosystem influence the composition and function of that particular community [8]. This phenomenon is known as “priority effects”, and its potential significance in the gut microbiota assembly process during early life has reached considerable attention in recent years [7, 9, 10]. Priority effects occur in the gut when initial colonizing species either pre-empt or modify the ecological niche, resulting in either inhibition or facilitation of later arriving species [7]. Several conditions make strong priority effects more likely to occur [11]. Priority effects are favoured when the early-arriving species exhibit a large effect on the environment (e.g. early-arriving species deplete the niche of specific nutrients), when late-arriving species have high environmental requirements (e.g. late-arriving species have high demands for survival and growth in the niche), and when early-arriving and late-arriving species exhibit a high niche overlap (e.g. closely related taxa and/or taxa competing for the same resources in the niche). Priority effects have been demonstrated in gnotobiotic mouse models with selected *Bacteroides* species [12] or mouse faecal communities [9], and in hospital-associated preterm infants [13] with limited microbial exposure, confined to mainly skin and hospital-associated microbes. However, the previously studied species-interactions may not necessarily reflect interactions in the gut of the healthy term infant, which is often dominated by *Bifidobacterium* species [14]. The role of priority effects in *Bifidobacterium* species community assembly has to our knowledge only been explored in vitro using batch fermentations [15], and has not been validated in continuous culture systems or animal models. Nor has infant gut microbiota data with sufficient longitudinal sampling and taxonomical resolution been applied to understand priority effects in the context of the term infant´s gut. In theory, priority effects should be affected by community assembly principles such as selection [7]. Breastfeeding is one of the strongest deterministic factors for infant gut microbiota species selection [16–18], in part due to its high content of human milk oligosaccharides (HMOs), which are selectively consumed in the infant gut by key members of the *Bifidobacterium* genus [19]. Especially *B. longum* subsp. *infantis* is an efficient HMO-consuming species associated with health benefits for the infant [20–22]. However, the importance of priority effects in *Bifidobacterium* community assembly, as well as the potential deterministic role of HMOs in this process both remain elusive.

To study *Bifidobacterium* community assembly, we took advantage of our previously established infant cohort [23], Copenhagen Infant Gut (CIG) comprising 25 healthy breast- and mixed fed infants longitudinally sampled from birth until 6 months of age (9–11 samplings per infant). We previously analysed the gut microbiota by 16 S rRNA gene amplicon sequencing and found, in accordance with others [24, 25], that *Bifidobacterium* was by far the most abundant genus (64.3% of all reads), representing mainly *B. longum* (38.5%), *B. breve* (9.1%), and *B. bifidum* (8.0%) as the three most abundant taxa in the dataset [23]. By use of species/subspecies specific qPCR, we further quantified the absolute abundance of *B. breve, B. bifidum*, *B. longum* subsp. *longum*, and *B. longum* subsp. *infantis* [23]. All infants were breastfed for at least four months, except CIG15, who shifted to formula milk around one month of age. Three infants received a single course of oral antibiotics (CIG05, CIG10 and CIG16), two infants were born pre-term by C-section (CIG08 and CIG09), and three infants displayed poor colonization (CIG07, CIG18 and CIG19) with these *Bifidobacterium* species (Supplementary Table 1) [23]. Given that antibiotics [26], pre-term birth [27], and high degree of formula exposure [28] can disrupt infant gut microbiota assembly, we did not consider these further in our analyses. We focused on the remaining 16 infants that were all breastfed, term and vaginally born and studied the longitudinal dynamics of *Bifidobacterium* species with respect to priority effects within each infant. We noticed that the aforementioned dominant *Bifidobacterium* species exhibited different abundance patterns across individuals (Fig. 1 and Supplementary Fig. 1). We found that *B. longum* subsp*. infantis* ended up vastly dominating the gut microbiota over time in 11 of the 16 infants (44% of the whole cohort), reaching a relative abundance of 84.9 ± 12.8% (mean ± s.d.) after efficiently colonizing these infants somewhere between age 14 to 100 days depending on the individual (Fig. 1). Apart from one infant that was solely dominated by *B. longum* subsp. *infantis* throughout the entire sampling period, almost all of the infants were initially dominated by other *Bifidobacterium* species or combinations thereof. Some infants were initially dominated by *B. breve* (Fig. 1a–c), others were dominated by *B. longum* subsp. *longum* (Fig. 1d, e), *B.* *bifidum* (Fig. 1f), or combinations of *B. longum* subsp. *longum*, *B. bifidum*, *B. breve*, *B. catenulatum* group, and *Bacteroides* species (Fig. 1g–j). Evidently, in the very first sample from these infants (age range 1–31 days), *B. longum* subsp. *infantis* was either not detected (below limit of detection; 2 × 10^2^ cells/g faeces), as for CIG03, CIG06 and CIG21, or present in counts below 10^4^ cells/g faeces as for CIG02, CIG04, CIG11, CIG22, CIG24 and CIG23. One exception was CIG01, where *B. longum* subsp. *infantis* reached above 10^7^ cells/g in the first sample (day 30), yet it was 100 fold lower in abundance compared to the dominating species *B. bifidum*. Thus, these data show that other *Bifidobacterium* species often colonize the breastfed infant’s gut before *B. longum* subsp. *infantis*, enabling them to initially dominate the community. However, this dominance is only transient as *B. longum* subsp. *infantis* eventually takes over. We also found that while CIG01 stopped being breastfed before the last sampling, *B. longum* subsp. *infantis* suddenly no longer dominated the community and other *Bifidobacterium* species reached higher absolute abundances (Fig. 1f), suggesting that *B. longum* subsp. *infantis* only dominates the community as long as the infant is primarily breastfed. In the case of CIG12, *B. longum* subsp. *infantis* consistently dominated throughout the entire sampling period (Fig. 1k), suggesting that this subspecies was the first *Bifidobacterium* colonizer in this particular infant. However, we cannot exclude the possibility that other *Bifidobacterium* species actually dominated before the first sample was collected (day 16), as seen with *B. breve* in CIG06 (Fig. 1c). Finally, we never detected *B. longum* subsp. *infantis* in two infants (CIG17 and CIG25), which were instead colonized mainly by *B. longum* subsp. *longum* alone or in combination with *B. breve* and *B. bifidum* (Supplementary Fig. 1a, b).

**Fig. 1:**
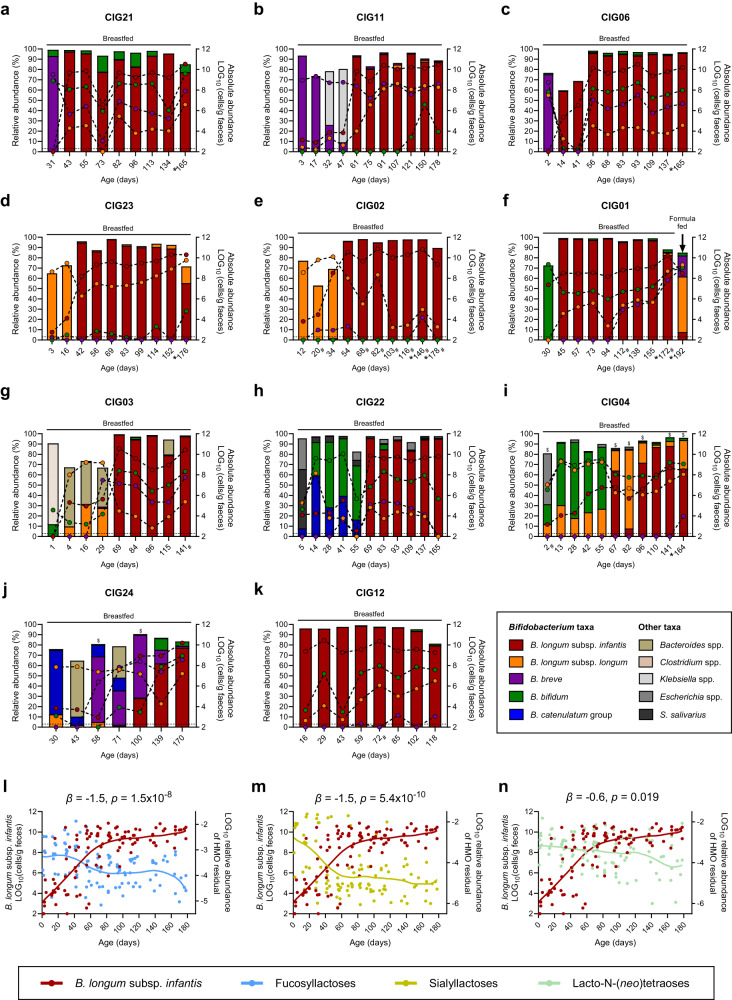
*B. longum* subsp. *infantis* eventually dominates the breastfed infant’s gut due to efficient utilization of human milk oligosaccharides. **a**–**k** Longitudinal relative abundance (bars) and absolute abundance (dots connected by dashed lines) of the major bacterial taxa detected in faeces of eleven full term, vaginally delivered, antibiotics naïve, breastfed infants from the Copenhagen Infant Gut cohort, as measured by 16 S rRNA gene amplicon sequencing (bars) and qPCR (dots), respectively. Only the abundant *Bifidobacterium* species, *B. longum* subsp. *longum*, *B. longum* subsp. *infantis, B. breve* and *B. bifidum* were quantified by qPCR. Dashed line illustrate the limit of detection (LOD) of the qPCR assay. *time points where solid foods have been consumed. ^#^time points where breastfeeding was supplemented with formula milk. ^$^Inconsistency between qPCR and 16 S rRNA gene amplicon sequence data regarding the dominant *Bifidobacterium* species. **l**, **m**, **n** Longitudinal absolute abundance of *B. longum* subsp. *infantis* and residual HMOs in faeces across all eleven infants displayed in panel (**a**–**k**) (the last sample from CIG01 was excluded due to cessation of breastfeeding). Statistical significance of the associations between *B. longum* subsp. *infantis* and (**l**) Fucosyllactoses, (**m**) Sialyllactoses and (**n**) Lacto-N-(*neo*)tetraoses were evaluated by linear mixed models, with *β* denoting the subject adjusted association coefficient. Locally weighted scatterplot smoothing (LOWESS) curves were fitted to the data points.

We then sought to couple these observations with in vivo HMO-utilization. We compared absolute abundance of *B. longum* subsp. *infantis* with the residual faecal levels of the major HMO structures (i.e. fucosyllactoses (2’FL and 3FL), sialyllactoses (3’SL and 6’SL), and lacto-N-(*neo*)tetraoses (LNT and LN*n*T)) in the eleven infants with dominant *B. longum* subsp. *infantis* colonization (Fig. 1a–k). The obvious bloom of *B. longum* subsp. *infantis* occurring within the first months of life in these infants coincided with a rapid decline in the faecal residuals of fucosyllactoses and sialyllactoses, whereas lacto-N-(*neo*)tetraoses were less prominently affected (Fig. 1l–n). These associations withstood adjustment of infant age and were, except for weaker associations with *B. bifidum*, not observed for the other *Bifidobacterium* species (Supplementary Table 2). This is largely consistent with the broad and efficient HMO-utilization capability of *B. longum* subsp*. infantis* strains enabling them to consume a range of fucosylated and sialylated HMOs [29–31], a more variable and less efficient HMO-utilization among *B. bifidum* strains [31, 32], and a limited capacity in *B. breve* and *B. longum* subsp*. longum* strains often confined to LNT and LN*n*T [33, 34]. However, in three infants (CIG13, CIG14 and CIG20), we did not observe a bloom in *B. longum* subsp. *infantis* over time, even though this subspecies was detected in low quantities throughout the sampling period. Instead, these infants became dominantly colonized with a combination of *B. breve* and *B. bifidum* (Supplementary Fig. 2c–e), which are known to be capable of cross-feeding HMOs efficiently [35, 36]. We speculate that this combination hindered *B. longum* subsp. *infantis* from taking over in these infants and/or that the *B. longum* subsp. *infantis* strains in these infants lack key genes to metabolize HMOs [37]. Indeed, in these three infants, faecal residuals of HMOs correlated negatively with the abundance of *B. breve*/*B. bifidum* and not *B. longum* subsp. *infantis* (Supplementary Table 2). Together, these data suggest that the initial *Bifidobacterium* colonizers are able to temporarily benefit from priority effects, but the strength of these priority effects are over time modified by breastfeeding selecting for efficient HMO-utilizers. Our data suggest that this is most commonly *B. longum* subsp. *infantis*, but it might also be other taxa or combinations thereof, as indicated in the three infants eventually dominated by *B. breve/B. bifidum*.

To demonstrate priority effects and validate the observations in our cohort with respect to key *Bifidobacterium* members, we designed an animal experiment, using sequential inoculation of the type strains of *B. longum* subsp*. infantis* DSM 20088 and *B. longum* subsp*. longum* DSM 20219 into germ free (GF) mice with or without supplying a 1.5% (w/v) mixture of HMOs (an equal mixture of 2’FL, 3FL, LNT, LN*n*T, 3’SL and 6’SL) into the drinking water as model of breastfeeding (Fig. 2a). We chose these strains given our observations of shifts in dominance between exactly these two subspecies within multiple infants (Fig. 1d, e, g, i), and since it is common that infants are initially colonized with maternal (vertically acquired) *B. longum* subsp. *longum* strains [38], but later colonized preferentially with (horizontally acquired) *B. longum* subsp. *infantis* strains [39]. Furthermore, a higher niche overlap would be expected between closely related taxa, such as strains of same species, facilitating priority effects. However, since *B. longum* subsp. *longum* versus *B. longum* subsp*. infantis* strains generally differ substantially in their HMO-degradation profiles [30], we would expect to be able to modify the strength of priority effects in the presence of HMOs. We first confirmed the differences in HMO-utilization between the type strains of these two subspecies by culturing them in vitro individually on 2’FL, 3FL, LNT, LN*n*T, 3’SL and 6’SL (Supplementary Fig. 2). *B. longum* subsp. *longum* DSM 20219 grew mainly on LNT and LN*n*T, whereas *B. longum* subsp*. infantis* DSM 20088 grew well on all six HMOs, which is highly consistent with the general picture of HMO-utilization reported on infant isolates of the two subspecies [31, 34]. Nonetheless, since it has been reported that a fraction of *B. longum* subsp. *longum* strains isolated from breastfed infants are capable of efficiently utilizing fucosyllactoses [31, 40], we cannot rule out that *B. longum* subsp. *longum* strains with HMO-utilization capability superior to the type strain may exist in our cohort. However, we did not find any significant associations between abundance of *B. longum* subsp. *longum* and faecal residuals of fucosyllactoses in the 11 *B. longum* subsp. *infantis* dominated infants (Fig. 1 and Supplementary Table 2). Together, this suggest that the HMO-utilization profiles of the type strains are well representing HMO-degradation capabilities of the strains found in the CIG infants.

**Fig. 2:**
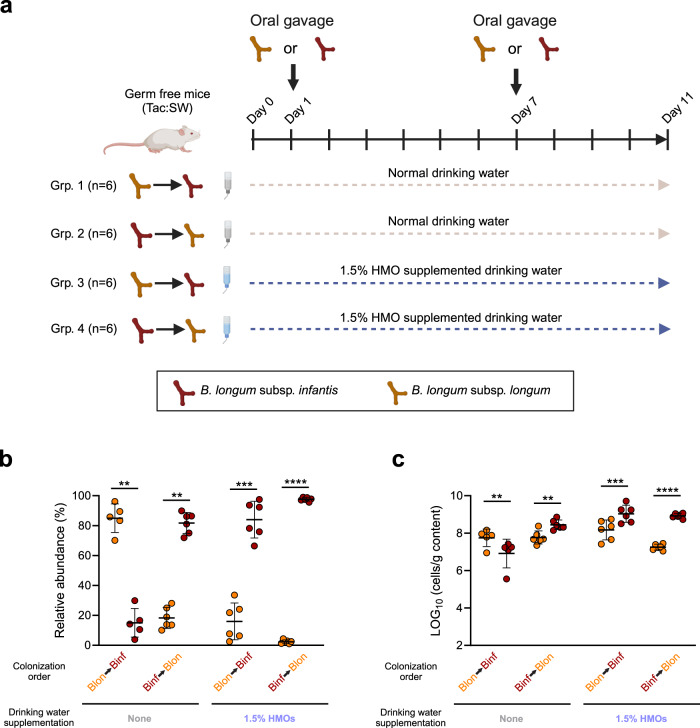
The strength of priority effects in the mouse gut are modified by human milk oligosaccharide supplementation. **a** Experimental design of the study. Germ free mice, consuming either normal drinking water or drinking water supplemented with HMOs, were sequentially colonized with *B. longum* subsp. *infantis* DSM 20088 and *B. longum* subsp. *longum* DSM 20219 or vice versa at day 1 and day 7 and caecal contents were sampled after euthanization at day 11. **b**, **c** Relative and absolute abundances of *B. longum* subsp. *infantis* DSM 20088 and *B. longum* subsp. *longum* DSM 20219 in caecal contents of the mice at day 11, quantified by subspecies-specific qPCR. Relative abundances were calculated by dividing the qPCR estimated counts of the given subspecies by the sum of the counts of the two and multiplying with hundred. Data represent mean ± s.d. and statistical significance was evaluated by two tailed paired *T*-tests. Data from one mouse in group 1 was excluded due to very poor *B. longum* subsp. *longum* colonization (counts < LLOQ of 20 copies per reaction in the qPCR assay).

We then divided twenty-four GF mice into four groups and kept them on drinking water with or without HMOs, starting 24 h before oral gavage with approximately 5 × 10^8^ CFU/ml of either *B. longum* subsp. *longum* DSM 20219 or *B. longum* subsp. *infantis* DSM 20088 on day 1. The mice were subsequently orally gavaged with approximately 5 × 10^8^ CFU/ml of the other strain on day 7 and euthanized on day 11 (Fig. 2a). Clear priority effects were observed when the mice consumed normal drinking water, with the order of arrival determining the relative and absolute abundance of the two species in caecal contents on day 11 (Fig. 2b, c). In contrast, when the mice consumed the HMO-supplemented drinking water, *B. longum* subsp. *infantis* dominated regardless of the colonization order. Notably, the dominance of *B. longum* subsp. *infantis* was most pronounced when it colonized first (Fig. 2b, c). These data demonstrate priority effects in vivo using closely related *Bifidobacterium* subspecies highly prevalent in the infant gut microbiota and show that the consumption of HMOs can strongly modify the strength of priority effects in an experimental setting, explaining the observations from our infant cohort.

Our results has important conceptual consequences for the way we view infant gut microbiota assembly as it underlines the importance of both order of colonization, and breastfeeding (HMOs) as a selective force. Considering that efficient HMO-utilizers such as *B. longum* subsp. *infantis* have been associated to lower prevalence of atopic disease [20], less gut inflammation [41], improved immune system development [22], and improved growth among malnourished infants [21], our insights emphasise the role of breastfeeding to modify the strength of priority effects in early life. Although *B. longum* subsp. *infantis* was highly abundant in 11 out of 25 of the studied Danish infants, this subspecies has been reported to be present at a very low prevalence, even in breastfed infants, in many Western populations, raising concerns about its possible extinction [39, 42]. However, clinical trials with oral supplementation of *B. longum* subsp. *infantis* EVC001 in breastfed neonates have demonstrated abundant and persistent colonization of this strain [43, 44]. Thus, in light of our observations, promoting and supporting breastfeeding remains a key priority, which may be complemented with early probiotic administration to ensure prominent gut colonization with key *Bifidobacterium* taxa during early infancy.

### Supplementary information

Supplementary information

## Data Availability

16 S rRNA gene amplicon sequencing data has been deposited in the Sequence Read Archive (SRA) under BioProject PRJNA554596.
