# Supplementary material for: Human milk oligosaccharides modify the strength of priority effects in the Bifidobacterium community assembly during infancy
Source: ISME J. 2023 Oct 10;17(12):2452–7. doi: 10.1038/s41396-023-01525-7 (PMC10689826; doi:10.1038/s41396-023-01525-7)
Supplement: Supplementary file 1 — Supplementary information [file 41396_2023_1525_MOESM1_ESM.docx]

**Supplementary information for “Human milk oligosaccharides modify the strength of priority effects in the *Bifidobacterium* community assembly during infancy”**

**Content**

**METHODS**

**SUPPLEMENTARY FIGURES**

**SUPPLEMENTARY TABLES**

**REFERENCES**

**METHODS**

***In vitro growth of Bifidobacterium strains on HMOs***

*B. longum* subsp. *longum* DSM 20219 and *B.* *longum* subsp. *infantis* DSM 20088 were cultivated on MRS agar plates with 0.05% (v/w) L-cysteine (MRSc) for 48h at 37°C anaerobically. Pre-cultures were prepared by inoculation of colony material into liquid MRSc, incubation for 20h with shake at 37°C anaerobically, before centrifugation at 10000 x g for 5 min, wash and re-suspension in prereduced sterile 0.9% saline water and dilution to OD_600nm_ = 0.05 in modified MRSc (mMRSc, prepared without addition of glucose) + 1% (w/v) carbon (C)-source (2’FL, 3FL, LNT, LN*n*T, 3’SL or 6’SL (Glycom A/S, Hørsholm, Denmark) or Lactose (Merck, 128951)) or without added C-source. Triplicate cultures were incubated at 37°C anaerobically for 48h before OD_600nm_ measurements.

***Preparation of cultures for oral gavage***

*B. longum* subsp. *longum* DSM 20219 and *B.* *longum* subsp. *infantis* DSM 20088 were cultivated on MRSc agar plates for 48h at 37°C anaerobically. Single colonies were dissolved in 5.0 mL pre-reduced MRSc broth and incubated for 20h at 37°C anaerobically with shake. The overnight (ON) cultures were washed (10000 x g, room temperature, 5 min) and resuspended using sterile pre-reduced PBS to OD_600nm_ = 2, giving a cell density of approximately 5x10^8^ CFU/ml. These washed cultures were used to orally gavage germ free (GF) mice at experimental day 1 and 7. The exact CFU counts in the inocula were found by plating the serially diluted PBS-washed cultures on MRSc agar after incubation anaerobically at 37°C for 48h.

***Animal experiment***

Twentyfour GF Swiss Webster mice (Tac:SW) were bred and housed within GF isolators (Scanbur, Karlslunde, Denmark) in type II makrolon cages (Techniplast, Varese, Italy) with bedding, nesting material, hiding place and a wooden block at the National Food Institute, Technical University of Denmark. The mice were fed an irradiated AIN-93G diet (Research diets, New Brunswick, NJ, US) *ad libitum* throughout the experimental period and the environment was maintained on a 12h light/12h dark cycle at a constant temperature of 22 ± 1 °C, with air humidity of 55 ± 5% relative humidity and change of air 50 times per hour. At 8-10 weeks of age the 24 mice were distributed into 4 separate isolators (4 groups containing each 4 males and 2 females), where they were single-cage housed and acclimatized for 6 days before intervention (at experimental day 0) with sterile filtered 1.5% HMOs (0.25% of each 2’FL, 3FL, LNT, LN*n*T, 3’SL and 6’SL) drinking water (group 3-4) or normal sterile drinking water (group 1-2) throughout the experiment. Faecal samples were collected daily from day 0 until termination. The GF status of mice prior to oral gavage was confirmed by inoculation of faeces from all groups separately into BHI broth (25°C and 37°C, aerobic incubation), mGAM broth (37°C, anaerobic incubation) and plating on blood agar (37°C, aerobic incubation) and evaluation after 24h and 2 weeks of incubation. At day 1 mice were orally gavaged with 100 ul PBS-washed ON culture containing approximately 5x10^8^ CFU/ml of *B. longum* subsp. *longum* DSM 20219 (group 1 and 3) or *B. longum* subsp. *infantis* DSM 20088 (group 2 and 4). This procedure was then repeated on day 7, however using the opposite strain compared to day 1. At day 11 all mice were anestisized in hypnorm/midazolam (0.1 ml/10g SC), terminal heart blood was collected and the mice were euthanized by cervical dislocation, before collection of gastrointestinal luminal content and tissue. Caecum content was homogenised 1:4 with sterile MilliQ water by vortexing and subjected to centrifugation (10000 x g, 5 min, 4°C), before collection of both pellets and supernatants in separate tubes, and snap freezing on dry ice and storage at -80°C until further processing. DNA was extracted from the thawed caecal pellets using the DNeasy PowerLyzer PowerSoil Kit (12855-100, Qiagen) and used for qPCR-based quantification of the *Bifidobacterium* strains. The animal experiment was approved by the Danish Animal Experiment inspectorate (License Number: 2020-15-0201-00484) and was overseen by the National Food Institute's in-house Animal Welfare Committee for animal care and use.

***Copenhagen infant gut cohort: sample collection and metadata***

As previously described [1] the Copenhagen Infant Gut (CIG) cohort includes 25 healthy infants (13 females and 12 males) from which faecal samples were collected at eleven distinct time points throughout the first 6 months of life (approximately at 0-2, 2-4, 4-6, 6-8, 8-10, 10-12, 12-14, 14-16, 16-20, 20-24 and 24-28 weeks of age). Parents collected fresh faecal samples from nappies into sterile faeces collection tubes (Sarstedt) and immediately stored them at –18 °C in a home freezer until transportation to the Technical University of Denmark where the samples were stored at –80 °C until sample preparation. Parents filled in a sheet with information about their child’s gender, term and mode of delivery, consumption of antibiotics, milk feeding patterns and timing of introduction to solid foods. The Data Protection Agency (18/02459) approved the study and the office of the Committees on Biomedical Research Ethics for the Capital Region of Denmark confirmed that the study was not notifiable according to the Act on Research Ethics Review of Health Research Projects (paragraph 1, subsection 4). Informed consent was obtained from all parents of infants participating in the study. In addition, parents of twins gave informed consent to publish data from the twins although the parents themselves would be able to identify their children using indirect identifiers. The parents did not receive any compensation.

***16S rRNA gene amplicon sequencing***

As previously published [1] we extracted DNA from 241 faecal samples originating from the 25 infants participating in the CIG study and performed 16S rRNA gene amplicon sequencing of the V3 region using the Ion PGM platform. Reads were de-multiplexed according to barcode and trimmed in CLC Genomic Workbench (v8.5. CLCbio, Qiagen, Aarhus, DK). Quality filtering, dereplication, OTU clustering, chimera filtering, mapping of reads to OTUs and generation of OTU tables was done according to the UPARSE pipeline [2]. In QIIME [3], OTU tables (nOTUs = 478) were filtered to include only OTUs with abundance across all samples above 0.005% of the total OTU counts (nOTUs = 145), ending up with 40156 ± 17614 (mean ± SD) reads. OTU relative abundances within samples were then estimated by total sum scaling. Taxonomy was assigned to the OTUs using the rdp classifier [4] with confidence threshold 0.5 and the GreenGenes database v13.8 [5]. Estimating species composition in the CIG cohort, the OTUs detected with identical taxonomy were collapsed and using a cutoff of average relative abundance of 0.1%, only 39 bacterial species/taxa remained, representing 97.5% of total community as described in Laursen *et al*.[1]. In order to investigate *Bifidobacterium* species composition OTUs sequences classified as *Bifidobacterium* according to the GreenGenes database v13.8 were filtered to remove low abundant OTUs (cutoff 0.1% of total *Bifidobacterium*) and the taxonomy of these resulting OTUs (nOTUs = 8) was confirmed by BLAST search against the 16S rRNA gene sequence database at NCBI. The top BLAST hit indicated species annotation and OTUs were collapsed into *Bifidobacterium* species (*B. longum*, *B.* *bifidum*, *B. breve, B. catenulatum* group, *B. adolescentis, B. scardovii, B. dentium, B. animalis/pseudolongum*) based on the top BLAST hit. Infant type *Bifidobacterium* spp. were defined as the summarised abundance of *B. longum*, *B. bifidum, B. breve* and *B. scardovii*. In this study relative abundances of *B. longum* subsp. *longum* and *B. longum* subsp. *infantis* were estimated by multiplying the relative abundance of *B. longum* (species level) from the 16S rRNA gene amplicon data with the absolute abundance (as quantified by qPCR, see below) of *B. longum* subsp. *infantis* or *B. longum* subsp. *longum*, divided by their summarized abundance. One sample (CIG24.11, taken at infant age 170 days) showed low quality sequence data and relative abundance of the *Bifidobacterium* species were instead estimated by qPCR, by normalizing qPCR-estimated counts to qPCR-estimated total bacterial load using universal primers targeting the 16S rRNA gene (see below).

***Quantitative PCR***

As described previously [1], absolute abundances of *B. longum* subsp. *longum*, *B.* *longum* subsp. *infantis*, *B. bifidum,* *B. breve,* as well as total bacterial load were estimated using DNA extracted from CIG infants faeces or caecal content of gnotobiotic mice by quantitative PCR, using species/subspecies specific or universal primers: *B. longum* subsp. *longum* (lon_0274_F: 5’-GAGGCGATGGTCTGGAAGTT -3’, lon_0274_R: 5’-CCACATCGCCGAGAAGATTC-3’, final concentration 0.75 µM each, annealing temperature 50°C), *B. longum* subsp. *infantis* (Blon0915F: 5’- CGTATTGGCTTTGTACGCATTT-3’, Blon0915R: 5’- ATCGTGCCGGTGAGATTTAC-3’, final concentration 0.75 µM each, annealing temperature 50°C), *B. bifidum* (BiBIF-1: 5’-CCACATGATCGCATGTGATTG-3’, BiBIF-2: 5’-CCGAAGGCTTGCTCCCAAA-3’, final concentration 0.5 µM each, annealing temperature 60°C), *B. breve* (B_bre-f: 5’-GCTCGTCGTTGCCGCCAAGGACGTT-3’. B_bre-r: 5’-ACAGAATGTACGGATCCTCGAGCACG-3’, final concentration 0.5 µM each, annealing temperature 72°C), and all bacteria (PBU: 5’-CCTACGGGAGGCAGCAG -3’. PBR: 5’- ATTACCGCGGCTGCTGG-3’, final concentration 0.2 µM each, annealing temperature 60°C). Each reaction was performed (in triplicates) with 5 µl PCR-grade water, 1.5 µl forward and reverse primer, 10 µl SYBR Green I Master 2X (LightCycler® 480 SYBR Green I Master, Roche) and 2 µl template DNA, in a total volume of 20 µl. Standard curves were generated from 10-fold serial dilutions of linearized plasmid (containing 10^8^ -10^1^ gene copies/µl), constructed by cloning of a PCR amplified 307bp fragment of the Blon0915 gene of *B. longum* subsp. *infantis* (DSM 20088), a 301bp fragment of the BL0274 gene of *B. longum* subsp. *longum* (DSM 20219) into a pCRII-Blunt-TOPO vector (Invitrogen) or 199bp fragment of the 16S rRNA gene (V3-region) of *E. coli* (ATCC 25922) into a pCR4-Blunt-TOPO vector (Invitrogen). For *B. bifidum* (16S rRNA gene) and *B. breve* (groEL gene), 10-fold serial dilutions of DNA (containing 10^7^ – 10^1^ gene copies/µl) extracted from pure cultures of the type strains (DSM 20456 and DSM 20213) were used for standard curves. Plates were run on the LightCycler® 480 Instrument II (Roche) with the program including 5 min pre-incubation at 95°C, followed by 45 cycles with 15 sec at 95°C, 15 sec at 50-72°C (depending on the primer) and 15 sec at 72°C and a subsequent melting curve analysis including 5 min at 95°C, 1 min at 65°C and continuous temperature increase (ramp rate 0.11 °C/s) until 98°C. Data were analysed with the LightCycler® 480 Software (v1.5) (Roche). Amplification was considered positive if, at least 2 out of 3 replicates had the correct melting curve compared to the standard (only for species/subspecies specific primers), and if the average Ct-value for the replicates gave rise to detection of at least one gene copy in the assay (based on linear regression of the known DNA quantities versus Ct-values in the corresponding standard curves). Lower limit of quantification was 20 copies per reaction for *B. breve*, *B. longum* subsp. *longum* and *B. longum* subsp. *infantis*, and 40 copies per reaction for *B. bifidum*. Based on faecal (infant cohort) and caecal (mouse study) weights, the concentrations of *Bifidobacterium* taxa or total number of bacterial cells per gram content were calculated, taking into account the gene copy number per genome of the species in question (Blon0915 gene in *B. longum* subsp. *infantis*, copy number per genome = 1; BL0274 gene in *B. longum* subsp. *longum*, copy number per genome = 1; groEL in *B. breve*, copy number per genome = 1 and 16S rRNA gene in *B. bifidum*, average copy number per genome = 3, 16S rRNA gene across all bacteria, average copy number per genome = 4). For the mouse study, relative abundances of *B. longum* subsp. *longum* and *B. longum* subsp. *infantis,* were calculated by dividing the counts of the given subspecies by the sum of the counts of the two and multiplying with hundred.

***HMO measurements***

As previously reported [1], faecal HMO residues (fucosyllactoses (2-‘FL and 3-FL), sialyllactoses (3’SL and 6’SL) and lacto-N-(*neo*)tetraoses (LNT and LN*n*T)) were measured by liquid chromatography mass spectrometry (LC-MS) in the faecal samples of the CIG cohort and confirmed by authentic standards. The HMO isomers could not be distinguished with the method applied due to identical retention times.

***Statistics***

Statistical analyses were performed in GraphPad Prism v10.0 (GraphPad Software, Inc. CA) or R v4.2 [6]. For the mouse experiment, paired T-tests (in GraphPad) were used to compare absolute and relative abundances of *Bifidobacterium longum* subspecies in caecal contents within individual mice from the same experimental group. For the CIG cohort infants, the Maaslin2 function within the R package maaslin2 (v1.0.0) [7] was used to model the LOG10 transformed qPCR estimated absolute abundances of the *Bifidobacterium* species versus LOG10 transformed relative abundance of faecal residuals of HMOs, including subject as a random effect, with otherwise default settings, except that total sum scaling normalization was not performed. In additional models infant age were incorporated as a fixed effect.

**SUPPLEMENTARY FIGURES**

**Supplementary Fig. 1.** **Breastfed infants displaying no or poor colonization with *B. longum* subsp. *infantis* are dominated by *B. longum* subsp. *longum* and/or *B. breve* and *B. bifidum*.** **a-e)** Longitudinal relative abundance (bars) and absolute abundance (dots connected by dashed lines) of the major bifidobacterial taxa detected in faeces of the five remaining full term, vaginally delivered, antibiotics naive breastfed infants from the Copenhagen Infant Gut cohort, as measured by 16S rRNA gene amplicon sequencing (bars) and qPCR (dots), respectively. Only the abundant *Bifidobacterium* species, *B. longum* subsp. *longum*, *B. longum* subsp. *infantis, B. breve,* and *B. bifidum* were quantified by qPCR. Dashed line illustrate the limit of detection (LOD) of the qPCR assay. *time points where solid foods have been consumed. ^$^Inconsistency between qPCR and 16S rRNA gene amplicon sequence data regarding the dominant *Bifidobacterium* species.

**Supplementary Fig. 2.** **Growth of the type strains of *B. longum* subspecies on human milk oligosaccharides *in vitro***. Growth of **a**) *B. longum* subsp. *longum* DSM 20219 **b**) and *B. longum* subsp. *infantis* DSM 20088 in modified MRSc medium (MRS prepared without addition of glucose but adding 0.05% (v/w) L-cysteine) containing 1% (w/v) carbon (C)-source of either 2’-fucosyllactose (2’FL), 3-fucosyllactose (3FL), Lacto-N-tetraose (LNT), Lacto-N-*neo*tetraose (LN*n*T), 3’-Sialyllactose (3’SL), 6’-Sialyllactose (6’SL), Lactose (positive control) or without added C-source (negative control).

**SUPPLEMENTARY TABLES**

| **Supplementary Table 1. Overview of the Copenhagen infant gut cohort infants.** | | | | | |  |  |
| --- | --- | --- | --- | --- | --- | --- | --- |
| **Infant ID** | **Relative abundance of Infant type *Bifidobacterium* species^1^** | **Antibiotics during the sampling period** | **Breastfeeding at least 4 months** | **Mode of delivery** | **Term of delivery** | **Suitable for study** | **Reason(s) for exclusion** |
| CIG01 | 93.60 | No | Yes | Vaginal | Full term | Yes |  |
| CIG02 | 87.24 | No | Yes | Vaginal | Full term | Yes |  |
| CIG03 | 61.84 | No | Yes | Vaginal | Full term | Yes |  |
| CIG04 | 84.64 | No | Yes | Vaginal | Full term | Yes |  |
| CIG05 | 38.45 | Yes (Oral antibiotics course before 3rd sampling) | Yes | Vaginal | Full term | No | Antibiotics |
| CIG06 | 88.46 | No | Yes | Vaginal | Full term | Yes |  |
| CIG07 | 8.52 | No | Yes | Vaginal | Full term | No | Low infant type *Bifidobacterium* abundance |
| CIG08 | 18.51 | No | Yes | C-section | Pre term | No | Preterm, C-section, Low infant type *Bifidobacterium* abundance |
| CIG09 | 13.64 | No | Yes | C-section | Pre term | No | Preterm, C-section, Low infant type *Bifidobacterium*  abundance |
| CIG10 | 6.21 | Yes (Oral antibiotics course before 5th sampling) | Yes | Vaginal | Full term | No | Antibiotics, Low infant type *Bifidobacterium*  abundance |
| CIG11 | 76.24 | No | Yes | Vaginal | Full term | Yes |  |
| CIG12 | 95.12 | No | Yes | Vaginal | Full term | Yes |  |
| CIG13 | 72.47 | No | Yes | Vaginal | Full term | Yes |  |
| CIG14 | 93.09 | No | Yes | Vaginal | Full term | Yes |  |
| CIG15 | 48.53 | No | No (stopped at 1 month of age) | Vaginal | Full term | No | Short breastfeeding duration |
| CIG16 | 54.27 | Yes (Oral antibiotics course before 6th sampling) | Yes | Vaginal | Full term | No | Antibiotics |
| CIG17 | 69.98 | No | Yes | Vaginal | Full term | Yes |  |
| CIG18 | 5.10 | No | Yes | Vaginal | Full term | No | Low infant type *Bifidobacterium*  abundance |
| CIG19 | 0.02 | No | Yes | Vaginal | Full term | No | Low infant type *Bifidobacterium*  abundance |
| CIG20 | 47.44 | No | Yes | Vaginal | Full term | Yes |  |
| CIG21 | 96.02 | No | Yes | Vaginal | Full term | Yes |  |
| CIG22 | 76.03 | No | Yes | Vaginal | Full term | Yes |  |
| CIG23 | 86.20 | No | Yes | Vaginal | Full term | Yes |  |
| CIG24 | 49.31 | No | Yes | Vaginal | Full term | Yes |  |
| CIG25 | 46.98 | No | Yes | Vaginal | Full term | Yes |  |
| ^1^Defined as the sum of *B. longum*, *B. breve,* *B. bifidum and B. scardovii* relative abundances (as in Laursen et al., 2021 Nature Microbiology), averaged across all sampling points. | | | | | | | |
|  |  |  |  |  |  |  |  |

| **Supplementary Table 2. Maaslin2 associations between qPCR estimated absolute abundance of *Bifidobacterium* taxa and relative abundance of HMO residuals in faeces from infants in the CIG cohort.** | | | | | | | | |
| --- | --- | --- | --- | --- | --- | --- | --- | --- |
|  |  |  |  |  |  |  |  |  |
| ***Model with HMO-type included as fixed effect and infant ID as random effect in the "B. infantis subset" (105 samples from 11 infants)*** | | | | | | | | |
| **Feature** | **Associated variable** | **Fixed effects** | **Random effects** | **β coefficient** | **s.d.** | **N** | **p-value** | **q-value** |
| *B. bifidum* | LN(*n*)T | LN(*n*)T | Subject ID | -0.630 | 0.168 | 105 | **3.00E-04** | **1.20E-03** |
| *B. longum subsp. infantis* | LN(*n*)T | LN(*n*)T | Subject ID | -0.609 | 0.256 | 105 | **0.019** | **0.038** |
| *B. longum subsp. longum* | LN(*n*)T | LN(*n*)T | Subject ID | -0.303 | 0.203 | 105 | 0.139 | 0.185 |
| *B. breve* | LN(*n*)T | LN(*n*)T | Subject ID | -0.168 | 0.173 | 105 | 0.335 | 0.335 |
| *B. longum subsp. infantis* | SL | SL | Subject ID | -1.479 | 0.216 | 105 | **5.39E-10** | **2.16E-09** |
| *B. bifidum* | SL | SL | Subject ID | -0.442 | 0.172 | 105 | **0.012** | **0.023** |
| *B. longum subsp. longum* | SL | SL | Subject ID | -0.105 | 0.202 | 105 | 0.604 | 0.604 |
| *B. breve* | SL | SL | Subject ID | -0.090 | 0.171 | 105 | 0.599 | 0.604 |
| *B. longum subsp. infantis* | FL | FL | Subject ID | -1.484 | 0.235 | 105 | **1.47E-08** | **5.88E-08** |
| *B. breve* | FL | FL | Subject ID | -0.284 | 0.194 | 105 | 0.147 | 0.293 |
| *B. longum subsp. longum* | FL | FL | Subject ID | -0.159 | 0.202 | 105 | 0.433 | 0.577 |
| *B. bifidum* | FL | FL | Subject ID | -0.094 | 0.226 | 105 | 0.677 | 0.677 |
|  |  |  |  |  |  |  |  |  |
| ***Model with age and HMO-type included as fixed effects and infant ID as random effect in the "B. infantis subset" (105 samples from 11 infants)*** | | | | | | | | |
| **Feature** | **Associated variable** | **Fixed effects** | **Random effects** | **β coefficient** | **s.d.** | **N** | **p-value** | **q-value** |
| *B. longum subsp. infantis* | Age | Age and LN(*n*)T | Subject ID | 1.802 | 0.178 | 105 | **1.03E-16** | **8.25E-16** |
| *B. breve* | Age | Age and LN(*n*)T | Subject ID | 0.575 | 0.157 | 105 | **4.14E-04** | **0.002** |
| *B. bifidum* | Age | Age and LN(*n*)T | Subject ID | 0.497 | 0.154 | 105 | **1.69E-03** | **4.52E-03** |
| *B. longum subsp. longum* | Age | Age and LN(*n*)T | Subject ID | -0.061 | 0.199 | 105 | 0.761 | 0.869 |
| *B. bifidum* | LN(*n*)T | Age and LN(*n*)T | Subject ID | -0.469 | 0.167 | 105 | **6.18E-03** | **1.24E-02** |
| *B. longum subsp. longum* | LN(*n*)T | Age and LN(*n*)T | Subject ID | -0.323 | 0.214 | 105 | 0.134 | 0.214 |
| *B. longum subsp. infantis* | LN(*n*)T | Age and LN(*n*)T | Subject ID | -0.120 | 0.190 | 105 | 0.529 | 0.705 |
| *B. breve* | LN(*n*)T | Age and LN(*n*)T | Subject ID | 0.018 | 0.171 | 105 | 0.918 | 0.918 |
| *B. longum subsp. infantis* | Age | Age and SL | Subject ID | 1.501 | 0.169 | 105 | **4.82E-14** | **3.85E-13** |
| *B. breve* | Age | Age and SL | Subject ID | 0.649 | 0.164 | 105 | **1.51E-04** | **4.02E-04** |
| *B. bifidum* | Age | Age and SL | Subject ID | 0.551 | 0.168 | 105 | **1.46E-03** | **2.92E-03** |
| *B. longum subsp. longum* | Age | Age and SL | Subject ID | -0.017 | 0.211 | 105 | 0.934 | 0.934 |
| *B. longum subsp. infantis* | SL | Age and SL | Subject ID | -0.846 | 0.179 | 105 | **7.38E-06** | **2.95E-05** |
| *B. bifidum* | SL | Age and SL | Subject ID | -0.189 | 0.180 | 105 | 0.295 | 0.394 |
| *B. longum subsp. longum* | SL | Age and SL | Subject ID | -0.112 | 0.223 | 105 | 0.616 | 0.704 |
| *B. breve* | SL | Age and SL | Subject ID | 0.201 | 0.176 | 105 | 0.256 | 0.394 |
| *B. longum subsp. infantis* | Age | Age and FL | Subject ID | 1.539 | 0.174 | 105 | **4.90E-14** | **3.92E-13** |
| *B. bifidum* | Age | Age and FL | Subject ID | 0.696 | 0.169 | 105 | **8.06E-05** | **2.61E-04** |
| *B. breve* | Age | Age and FL | Subject ID | 0.578 | 0.166 | 105 | **7.64E-04** | **1.53E-03** |
| *B. longum subsp. longum* | Age | Age and FL | Subject ID | -0.008 | 0.211 | 105 | 0.970 | 0.970 |
| *B. longum subsp. infantis* | FL | Age and FL | Subject ID | -0.821 | 0.202 | 105 | **9.77E-05** | **2.61E-04** |
| *B. longum subsp. longum* | FL | Age and FL | Subject ID | -0.098 | 0.250 | 105 | 0.696 | 0.928 |
| *B. breve* | FL | Age and FL | Subject ID | 0.020 | 0.204 | 105 | 0.921 | 0.970 |
| *B. bifidum* | FL | Age and FL | Subject ID | 0.203 | 0.207 | 105 | 0.328 | 0.525 |
|  |  |  |  |  |  |  |  |  |
| ***Model with HMO-type included as fixed effect and infant ID as random effect in the "B. breve/B. bifidum subset" (26 samples from 3 infants)*** | | | | | | | | |
| **Feature** | **Associated variable** | **Fixed effects** | **Random effects** | **β coefficient** | **s.d.** | **N** | **p-value** | **q-value** |
| *B. breve* | LN(*n*)T | LN(*n*)T | Subject ID | -2.072 | 0.509 | 26 | **4.68E-04** | **1.87E-03** |
| *B. bifidum* | LN(*n*)T | LN(*n*)T | Subject ID | -1.633 | 0.443 | 26 | **1.22E-03** | **2.44E-03** |
| *B. longum subsp. longum* | LN(*n*)T | LN(*n*)T | Subject ID | -0.573 | 0.308 | 26 | 0.076 | 0.101 |
| *B. longum subsp. infantis* | LN(*n*)T | LN(*n*)T | Subject ID | -0.250 | 0.202 | 26 | 0.228 | 0.228 |
| *B. breve* | SL | SL | Subject ID | -1.692 | 0.513 | 26 | **3.19E-03** | **1.28E-02** |
| *B. bifidum* | SL | SL | Subject ID | -0.753 | 0.497 | 26 | 0.144 | 0.191 |
| *B. longum subsp. longum* | SL | SL | Subject ID | -0.638 | 0.290 | 26 | **3.74E-02** | 0.075 |
| *B. longum subsp. infantis* | SL | SL | Subject ID | 0.056 | 0.197 | 26 | 0.780 | 0.780 |
| *B. bifidum* | FL | FL | Subject ID | -0.272 | 0.500 | 26 | 0.592 | 0.652 |
| *B. longum subsp. longum* | FL | FL | Subject ID | -0.194 | 0.305 | 26 | 0.530 | 0.652 |
| *B. longum subsp. infantis* | FL | FL | Subject ID | -0.168 | 0.184 | 26 | 0.370 | 0.652 |
| *B. breve* | FL | FL | Subject ID | 0.274 | 0.599 | 26 | 0.652 | 0.652 |
|  |  |  |  |  |  |  |  |  |
| ***Model with age and HMO-type included as fixed effect and infant ID as random effect in the "B. breve/B. bifidum subset" (26 samples from 3 infants)*** | | | | | | | | |
| **Feature** | **Associated variable** | **Fixed effects** | **Random effects** | **β coefficient** | **s.d.** | **N** | **p-value** | **q-value** |
| *B. breve* | Age | Age and LN(*n*)T | Subject ID | 1.705 | 0.358 | 26 | **9.85E-05** | **7.88E-04** |
| *B. bifidum* | Age | Age and LN(*n*)T | Subject ID | 1.179 | 0.369 | 26 | **4.24E-03** | **8.81E-03** |
| *B. longum subsp. longum* | Age | Age and LN(*n*)T | Subject ID | 0.826 | 0.261 | 26 | **4.41E-03** | **8.81E-03** |
| *B. longum subsp. infantis* | Age | Age and LN(*n*)T | Subject ID | -0.023 | 0.201 | 26 | 0.912 | 0.912 |
| *B. breve* | LN(*n*)T | Age and LN(*n*)T | Subject ID | -1.286 | 0.404 | 26 | **4.23E-03** | **8.81E-03** |
| *B. bifidum* | LN(*n*)T | Age and LN(*n*)T | Subject ID | -1.067 | 0.415 | 26 | **1.73E-02** | **2.77E-02** |
| *B. longum subsp. infantis* | LN(*n*)T | Age and LN(*n*)T | Subject ID | -0.260 | 0.226 | 26 | 0.262 | 0.349 |
| *B. longum subsp. longum* | LN(*n*)T | Age and LN(*n*)T | Subject ID | -0.226 | 0.289 | 26 | 0.443 | 0.506 |
| *B. breve* | Age | Age and SL | Subject ID | 1.903 | 0.476 | 26 | **5.90E-04** | **2.36E-03** |
| *B. bifidum* | Age | Age and SL | Subject ID | 1.932 | 0.455 | 26 | **3.81E-04** | **2.36E-03** |
| *B. longum subsp. longum* | Age | Age and SL | Subject ID | 0.854 | 0.296 | 26 | **8.37E-03** | **2.23E-02** |
| *B. longum subsp. infantis* | Age | Age and SL | Subject ID | 0.176 | 0.233 | 26 | 0.458 | 0.562 |
| *B. breve* | SL | Age and SL | Subject ID | -0.510 | 0.500 | 26 | 0.318 | 0.509 |
| *B. longum subsp. longum* | SL | Age and SL | Subject ID | -0.096 | 0.309 | 26 | 0.760 | 0.760 |
| *B. longum subsp. infantis* | SL | Age and SL | Subject ID | 0.170 | 0.244 | 26 | 0.492 | 0.562 |
| *B. bifidum* | SL | Age and SL | Subject ID | 0.527 | 0.464 | 26 | 0.274 | 0.509 |
| *B. breve* | Age | Age and FL | Subject ID | 2.267 | 0.411 | 26 | **1.69E-05** | **1.36E-04** |
| *B. bifidum* | Age | Age and FL | Subject ID | 1.773 | 0.373 | 26 | **1.03E-04** | **4.10E-04** |
| *B. longum subsp. longum* | Age | Age and FL | Subject ID | 1.020 | 0.232 | 26 | **2.41E-04** | **6.43E-04** |
| *B. longum subsp. infantis* | Age | Age and FL | Subject ID | 0.125 | 0.192 | 26 | 0.523 | 0.523 |
| *B. bifidum* | FL | Age and FL | Subject ID | -0.721 | 0.373 | 26 | 0.066 | 0.113 |
| *B. longum subsp. longum* | FL | Age and FL | Subject ID | -0.441 | 0.232 | 26 | 0.070 | 0.113 |
| *B. breve* | FL | Age and FL | Subject ID | -0.291 | 0.411 | 26 | 0.487 | 0.523 |
| *B. longum subsp. infantis* | FL | Age and FL | Subject ID | -0.198 | 0.192 | 26 | 0.316 | 0.421 |

**REFERENCES**

1. Laursen MF, Sakanaka M, von Burg N, Mörbe U, Andersen D, Moll JM, et al. *Bifidobacterium* species associated with breastfeeding produce aromatic lactic acids in the infant gut. *Nat Microbiol* 2021; **6**: 1367–1382.

2. Edgar RC. UPARSE: highly accurate OTU sequences from microbial amplicon reads. *Nat Methods* 2013; **10**: 996–8.

3. Caporaso JG, Kuczynski J, Stombaugh J, Bittinger K, Bushman FD, Costello EK, et al. QIIME allows analysis of high-throughput community sequencing data. *Nat Methods* 2010; **7**: 335–6.

4. Wang Q, Garrity GM, Tiedje JM, Cole JR. Naive Bayesian classifier for rapid assignment of rRNA sequences into the new bacterial taxonomy. *Appl Environ Microbiol* 2007; **73**: 5261–7.

5. Desantis TZ, Hugenholtz P, Larsen N, Rojas M, Brodie EL, Keller K, et al. Greengenes, a Chimera-Checked 16S rRNA Gene Database and Workbench Compatible with ARB. *Appl Enviromental Microbiol* 2006; **72**: 5069–5072.

6. R Core Team. R: A language and environment for statistical computing. 2013.

7. Mallick H, Rahnavard A, McIver LJ, Ma S, Zhang Y, Nguyen LH, et al. Multivariable association discovery in population-scale meta-omics studies. *PLOS Comput Biol* 2021; **17**: 1–27.
